# Exosomes derived from pioglitazone-pretreated MSCs accelerate diabetic wound healing through enhancing angiogenesis

**DOI:** 10.1186/s12951-021-00894-5

**Published:** 2021-05-21

**Authors:** Yiqiang Hu, Ranyang Tao, Lang Chen, Yuan Xiong, Hang Xue, Liangcong Hu, Chenchen Yan, Xudong Xie, Ze Lin, Adriana C. Panayi, Bobin Mi, Guohui Liu

**Affiliations:** 1 Department of Orthopaedics, Union Hospital, Tongji Medical College, Huazhong University of Science and Technology, Wuhan, 430022 China; 2 Hubei Province Key Laboratory of Oral and Maxillofacial Development and Regeneration, Wuhan, 430022 China; 3 Department of Plastic Surgery, Brigham and Womens Hospital, Harvard Medical School, Boston, MA 02215 USA

**Keywords:** Exosomes, Mesenchymal stem cells, Pioglitazone, Diabetic wound, Angiogenesis

## Abstract

**Background:**

Enhanced angiogenesis can promote diabetic wound healing. Mesenchymal stem cells (MSCs)-derived exosomes, which are cell-free therapeutics, are promising candidates for the treatment of diabetic wound healing. The present study aimed to investigate the effect of exosomes derived from MSCs pretreated with pioglitazone (PGZ-Exos) on diabetic wound healing.

**Results:**

We isolated PGZ-Exos from the supernatants of pioglitazone-treated BMSCs and found that PGZ-Exos significantly promote the cell viability and proliferation of Human Umbilical Vein Vascular Endothelial Cells (HUVECs) injured by high glucose (HG). PGZ-Exos enhanced the biological functions of HUVECs, including migration, tube formation, wound repair and VEGF expressionin vitro. In addition, PGZ-Exos promoted the protein expression of p-AKT, p-PI3K and p-eNOS and suppressed that of PTEN. LY294002 inhibited the biological function of HUVECs through inhibition of the PI3K/AKT/eNOS pathway. In vivo modeling in diabetic rat wounds showed that pioglitazone pretreatment enhanced the therapeutic efficacy of MSCs-derived exosomes and accelerated diabetic wound healing via enhanced angiogenesis. In addition, PGZ-Exos promoted collagen deposition, ECM remodeling and VEGF and CD31 expression, indicating adequate angiogenesis in diabetic wound healing.

**Conclusions:**

PGZ-Exos accelerated diabetic wound healing by promoting the angiogenic function of HUVECs through activation of the PI3K/AKT/eNOS pathway. This offers a promising novel cell-free therapy for treating diabetic wound healing.

**Graphic abstract:**

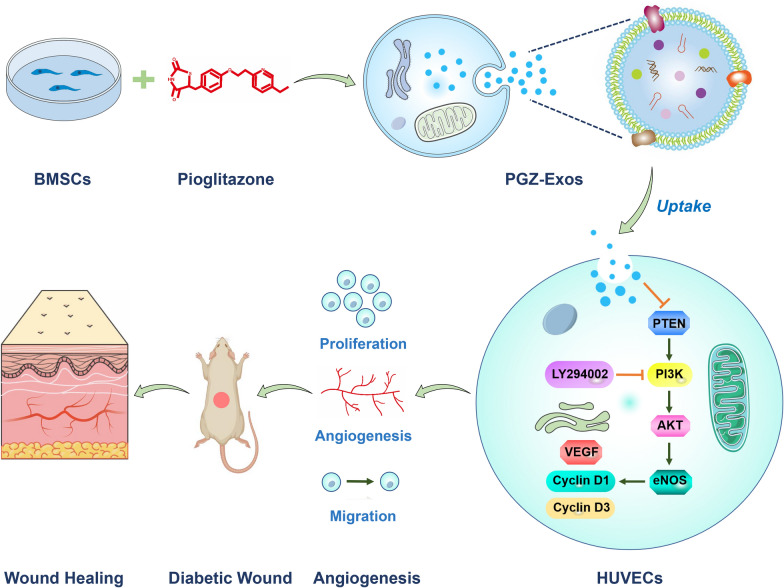

## Background

Complications of diabetes, such as recalcitrant wounds, are a significant worldwide cause of disability and mortality [1,3]. Diabetic wounds can have extensive negative repercussions on the quality of life of patients and result in great psychological distress [4, 5]. Current therapies include dressing changes and surgical debridement, but outcomes are not yet satisfactory [6, 7]. Vascular dysfunction caused by hyperglycemia results in insufficient blood supply to the wound surface, and is an important underlying reason for diabetic wound healing failure [8, 9]. Accumulating evidence has supported that enhanced angiogenesis can improve the delivery of nutrients and oxygen to the wound sites, which can accelerate wound healing [10, 11]. An effective strategy that promotes wound angiogenesis can accelerate wound healing and has the potential to revolutionize the treatment of patients with diabetes [12, 13].

Exosomes, extracellular vesicles with a diameter between 30 and 150 nm, can transport proteins and growth factors to target cells where they exert different effects [14, 15]. Bone marrow-derived MSCs (BMSCs) possess many advantages, including easier culturing, proliferation, isolation and purification, rendering them convenient for clinical application [16, 17]. Many researchers have reported on the clinical use of bone marrow-derived MSCs in the treatment of diseases, including cardiac disease [18], pulmonary disease [19], and osteoarthritis [20]. Recent research identified that exosomes derived from BMSCs can significantly promote the neovascularization and regenerative capacity of promoting tissue repair [21, 22]. Compared with MSCs, exosomes possess advantageous properties, including good stability and low immunogenicity [23, 24]. Recent research has reported that exosomes derived from MSCs have cardioprotective effects by inhibiting cardiomyocyte apoptotic injury [25]. BMSCs-derived exosomes have been shown to stimulate the proliferation and osteogenic differentiation of BMSCs to promote new bone regeneration and neovascularization in bone defects [26]. Therefore, exosomes are a cell-free therapeutic that may be safer than direct MSCs therapy and provide a potential modality for tissue regeneration [27, 28]. Based on these findings, exosomes may serve as a promising candidate to promote angiogenesis in wound healing.

Previous studies have reported that preconditioning of MSCs with various pretreatments, such as drugs, cytokines and physical factors is an effective approach to enhance the biological activities and function of MSCs in tissue regeneration [29, 30]. Recently, studies have demonstrated that pretreated MSCs exhibit enhanced paracrine effects. For instance, exosomes derived from hypoxia-preconditioned adipose-derived MSCs possess a higher capacity to enhance angiogenesis and promote graft survival compared with exosomes from untreated MSCs [31]. Exosomes derived from dimethyloxaloylglycine-pretreated MSCs enhance bone regeneration through angiogenic effects [32]. Pioglitazone, a peroxisome proliferator-activated receptor activator, is a common drug used in the treatment of diabetes. Studies have suggested that pioglitazone may play a potential protective role in inflammation and oxidative stress [33, 34]. Previous studies have reported that MSCs pretreated with pioglitazone can significantly improve the efficiency of cardiomyogenic transdifferentiation and cardiac function to enhance the efficacy of MSCs transplantation [35]. However, the effect of pioglitazone on exosomes derived from BMSCs and the possible underlying mechanisms have not yet been studied in diabetic wound healing.

Herein, our research aimed to explore the effect of exosomes derived from BMSCs pretreated with pioglitazone on the biological function of HUVECs under hyperglycemic conditions. We investigate the role of the PI3K/AKT/eNOS pathway in PGZ-Exos-treated diabetic wounds to assess the therapeutic effect in diabetic wound healing.

## Results

### Characterization of BMSCs-derived exosomes

**Fig. 1:**
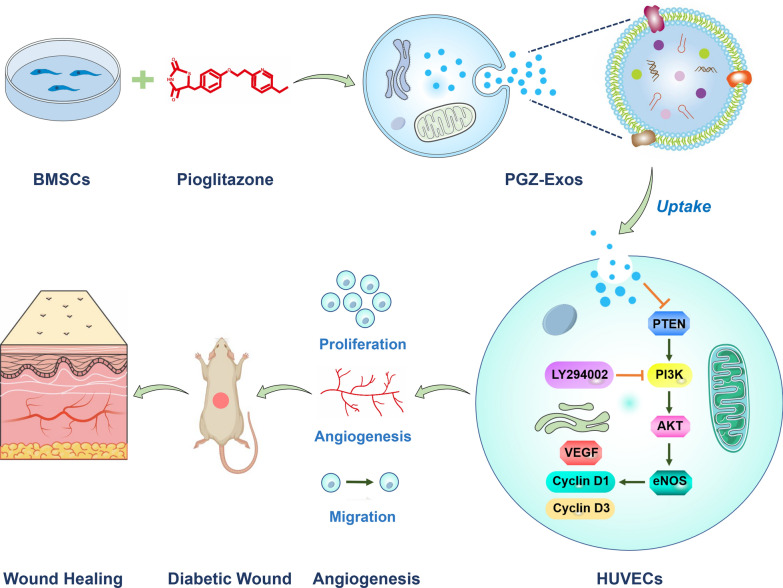
Schematic diagram of PGZ-Exos accelerating diabetic wound repair through enhancing angiogenesis by activation of the PI3K/AKT/eNOS pathway

**Fig. 2:**
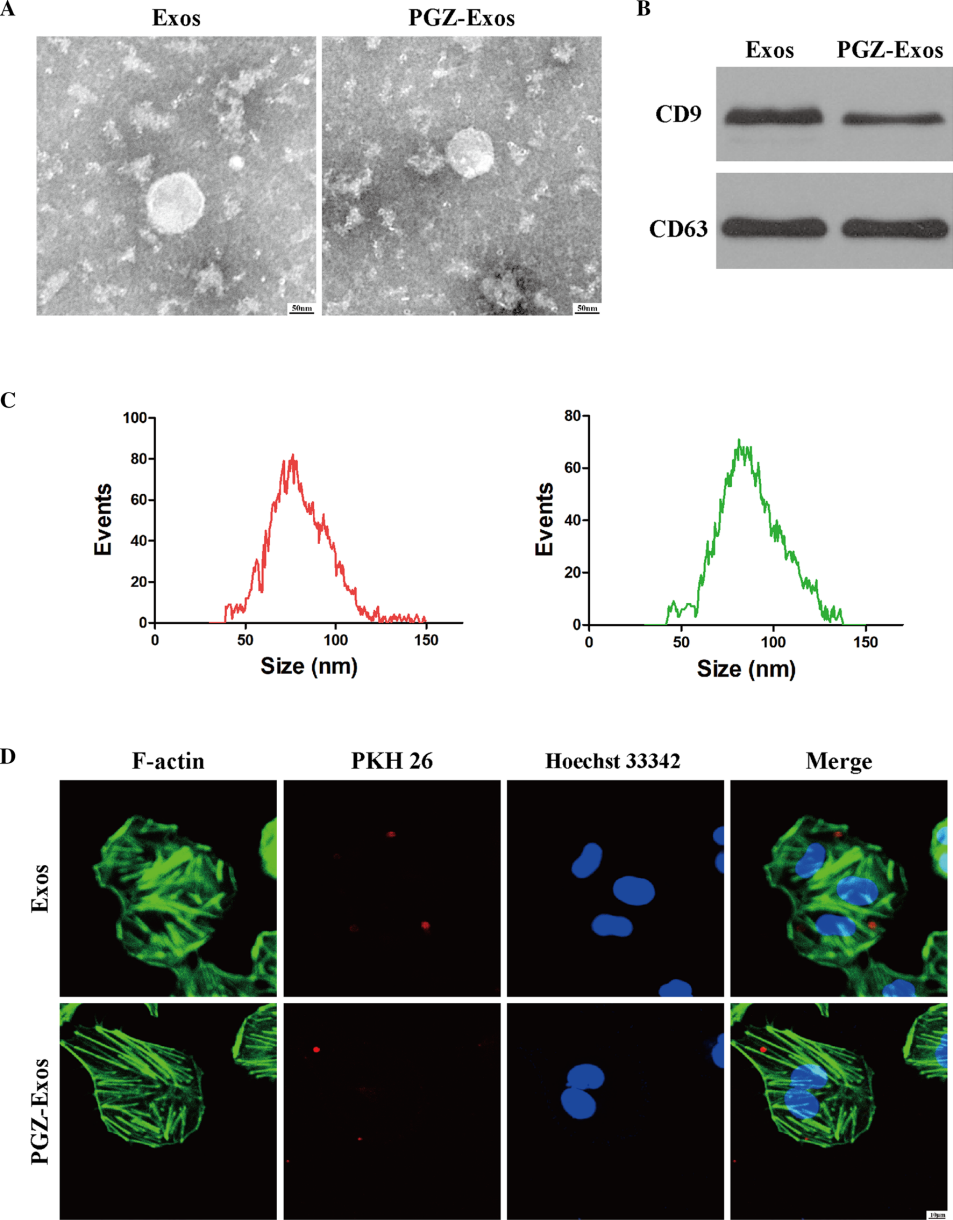
Characterization of BMSCs-derived exosomes. **A** The morphology of the exosomes and PGZ-Exos was visualized with TEM. Scale bar: 50 nm. **B** The exosome marker proteins CD9 and CD63 in the exosomes and PGZ-Exos were detected with western blotting. **C** The size distribution of exosomes and PGZ-Exos was examined with NTA. **D** PKH26-labeled exosomes and PGZ-Exos uptake by HUVECs was investigated with laser scanning confocal microscopy. The cytoskeleton, exosomes and cell nucleus are stained green, red, and blue respectively. Scale bar: 10 m

To explore the effect of PGZ-Exos on diabetic wounds healing (Fig. 1), the supernatants of BMSCs and pioglitazone-treated BMSCs were collected to isolate exosomes and PGZ-Exos, respectively via ultracentrifugation. We found, through assessment of TEM images, that the exosomes and PGZ-Exos contained typical exosomal structures, including homogeneous, spherical, and membrane vesicles (Fig. 2A). Exosomal surface markers were detected with western blotting. The results demonstrated that both the exosomes and PGZ-Exos isolated from BMSCs expressed exosomal markers, such as CD9 and CD63 (Fig. 2B). We measured the size distribution with NTA and found that the exosomes and PGZ-Exos mainlyranged from 50 to 120 nm (Fig. 2C). These data showed that morphology, protein and particle size were similar between exosomes and PGZ-Exos. The pioglitazone pretreatment had no impact on exosomal characteristicsfrom BMSCs. To investigate exosome and PGZ-Exos uptake by HUVECs, we labeled the exosomes and PGZ-Exos with PKH26 and co-cultured these with HUVECs finding that both the exosomes and PGZ-Exos were endocytosed by HUVECs (Fig. 2D).

### PGZ-exos protect against HG-induced inhibition of HUVECs viability and cell proliferation

**Fig. 3:**
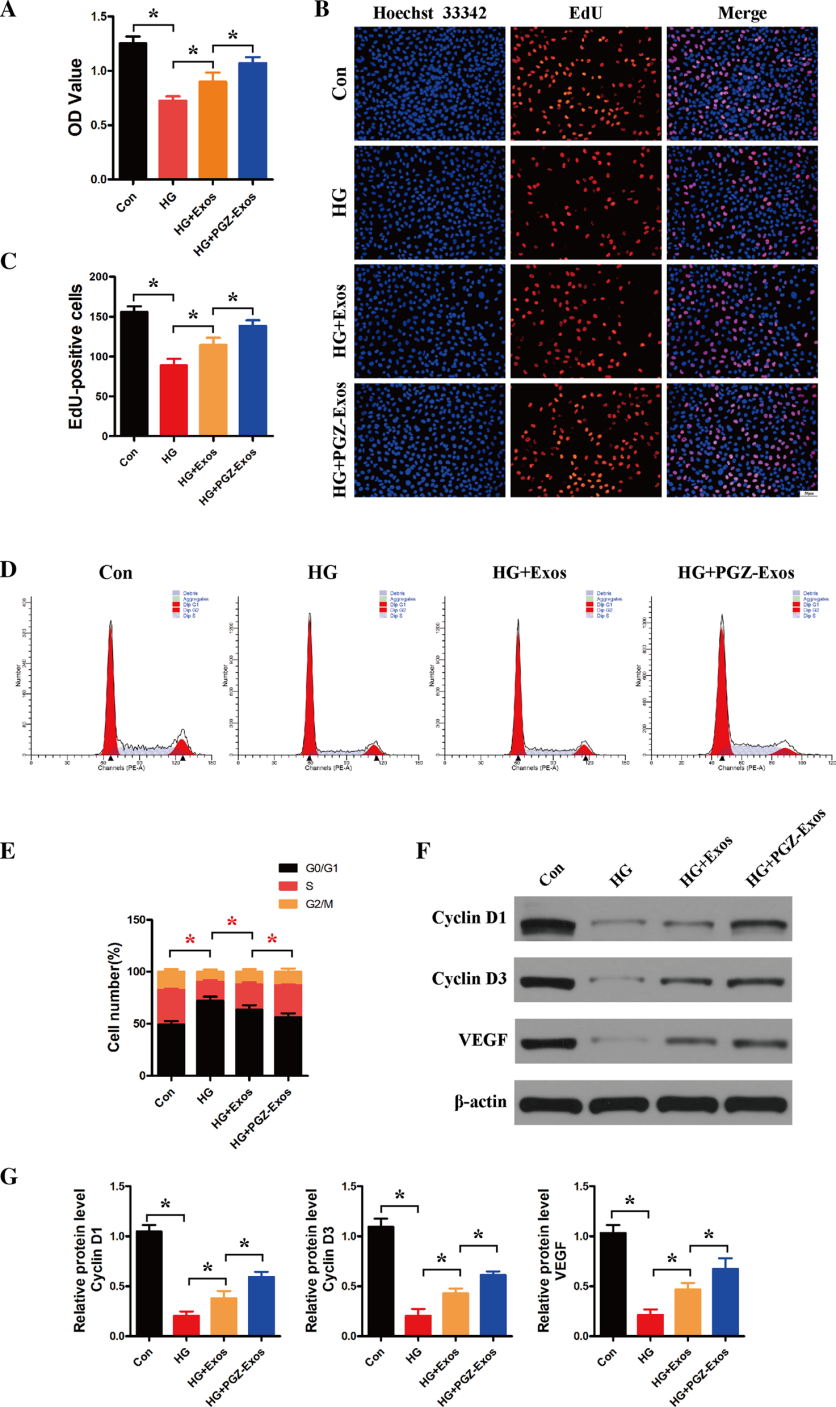
PGZ-exos protected against HG-induced inhibition of HUVECs viability and cell proliferation. **A** CCK8 assay was performed to assess HUVECs viability after treatment with HG and exosomes. **B**, **C** EdU incorporation assay was used to evaluate HUVECs proliferation. Scale bar: 50 m. **D**, **E** Flow cytometry was used to assess the cell cycle distribution of the HUVECs. **F**, **G** Western blotting was used to investigate the protein expression of Cyclin D1, Cyclin D3 and VEGF. Data are presented as meansSD from three independent experiments. *P<0.05 versus the control (con) group

A CCK-8 assay was performed to assess the PGZ-Exos effect on HUVECs viability. The results indicated that HG inhibited the viability of HUVECs while exosomes and PGZ-Exos protected against this effect, with PGZ-Exos exhibiting a stronger protective effect than exosomes (Fig. 3A). In addition, we used an EdU incorporation assay to detect HUVECs proliferation. The number of EdU-positive cells was higher in the exosome and PGZ-Exos groups compared with the HG group, with the PGZ-Exos group have the most EdU-positive cells (Fig. 3B, C). We further assessed the effect of exosomes and PGZ-Exos on the HUVECs cell cycle and found that both the exosomes and PGZ-Exos significantly increased the proportion of cells entering the S phase. The proportion of cells entering the S phase in PGZ-Exos was higher than that of the exosome group (Fig. 3D, E). Exosomes and PGZ-Exos significantly promoted the expression of the proliferation-related proteins Cyclin D1 and Cyclin D3 which had been inhibited by HG treatment. Higher Cyclin D1 and Cyclin D3 expression was seen in the PGZ-Exos group. PGZ-Exos significantly increased the expression of VEGF inducing angiogenesis and granulation tissue formation in wound healing compared with the HG and exosome treated groups (Fig. 3F, G). Taken together, these results indicate that PGZ-exos protect against the HG-induced inhibition of HUVEC viability and cell proliferation.

### PGZ-exos protect against HG-induced inhibition of the angiogenic ability of HUVECs

**Fig. 4:**
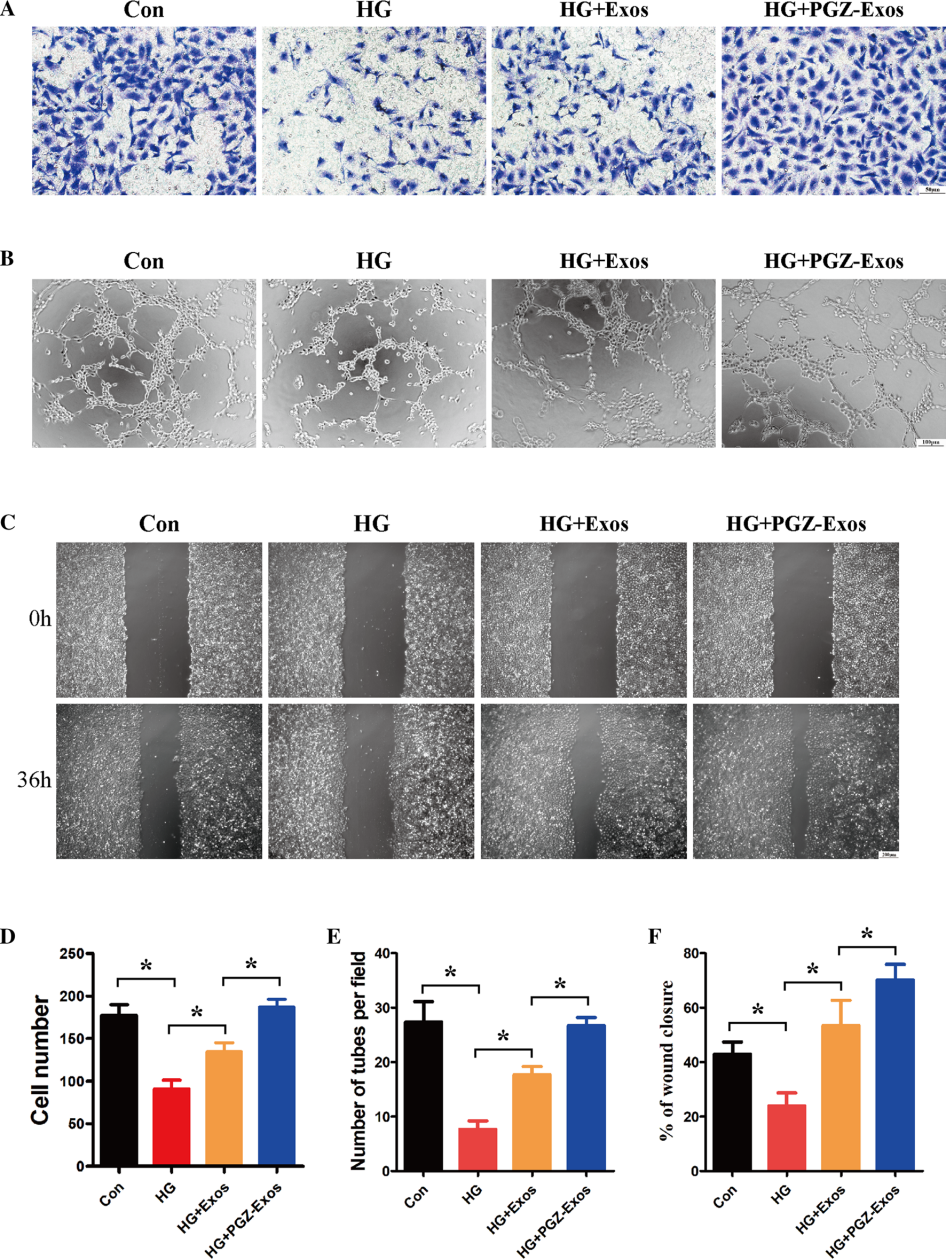
PGZ-exos enhanced the angiogenic ability of HUVECs inhibited by HG. **B** A transwell assay was used to assess the cell migration of HUVECs. Scale bar: 50 m. **B** A tube formation assay was performed to visualize the cell capillary network formation of HUVECs. Scale bar: 100 m. **C** In vitro wound healing assay of the HUVECs. Scale bar: 200 m. **D** Quantitative analysis of the number of migrating cells in the four groups. **E** Quantitative analysis of tube formation in the four groups. **F** Quantitative analysis of the rate of wound closure in the four groups. Data are presented as meansSD from three independent experiments. *P<0.05 versus the control (con) group

To assess the effect of PGZ-Exos on HUVECs migration, we performed a transwell migration assay. Our results revealed that both exosomes and PGZ-Exos increased the number of HUVECs migrating which hadbeen inhibited by HG, with PGZ-Exos exhibiting a stronger effect compared to exosomes group (Fig. 4A). Tube formation on matrigel was performed to evaluate the capillary network formation of HUVECs. The results demonstrated that the exosomes enhanced the tube formation ability of HUVECs under HG conditions, while PGZ-Exos exhibited the strongest improvement of tube formation (Fig. 4B). To investigate the effect of PGZ-Exos on wound healing in vitro, we performed a wound healing assay. Our results illustrated that HG inhibited wound healing in vitro, while exosomes and PGZ-Exos rescued healing. PGZ-Exos exhibited the strongest promotion effect in in vitro wound healing (Fig. 4C). These data indicated that PGZ-exos rescued the angiogenic ability of HUVECs which had been suppressed by HG (Fig. 4D-F).

### PGZ-Exos promoted angiogenesis by activating the PI3K/AKT/eNOS pathway

**Fig. 5:**
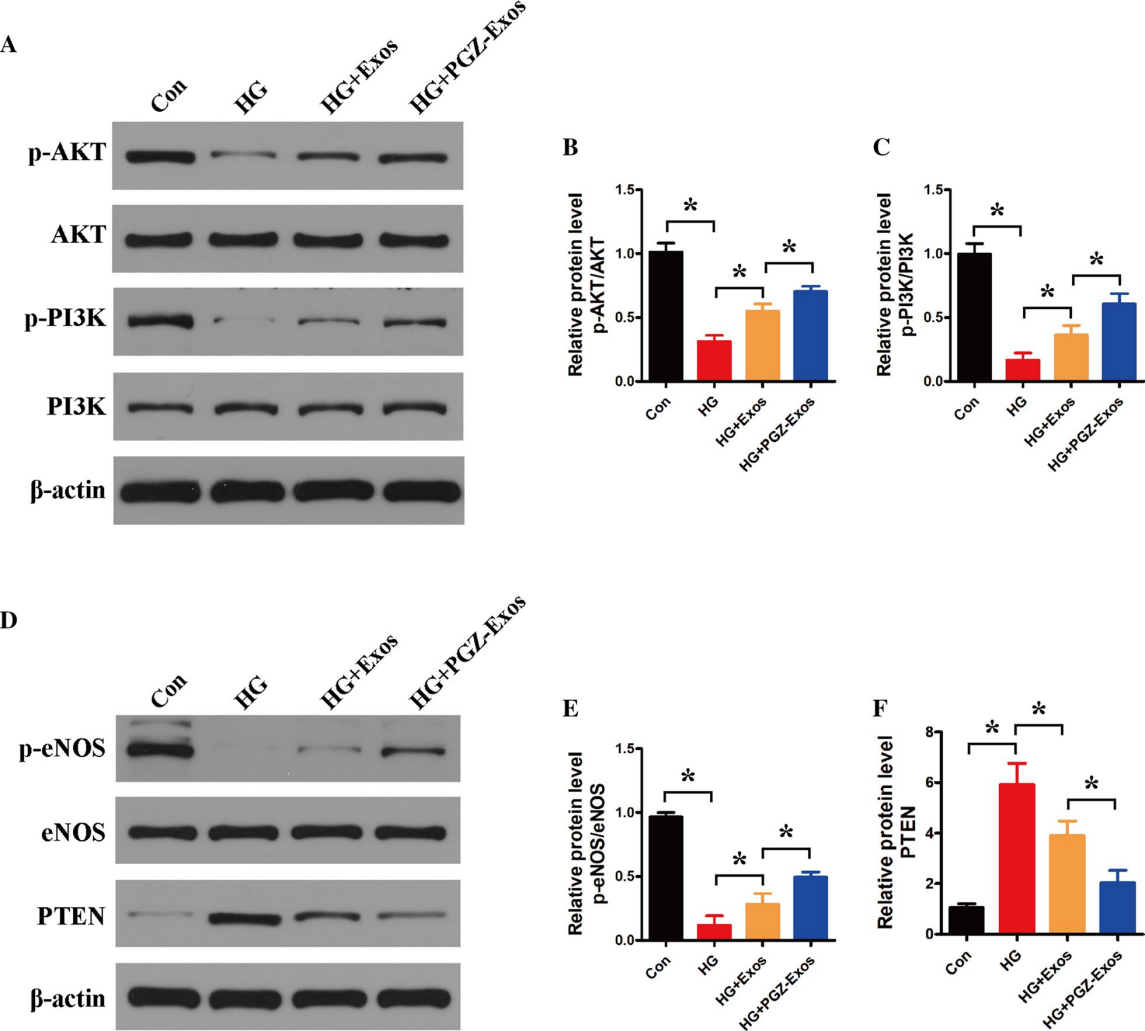
PGZ-Exos promoted the angiogenic ability of HUVECs through activation of the PI3K/AKT/eNOS pathway. **A** Western blotting showing the protein expression of AKT, p-AKT, PI3K and p-PI3K. **B** Quantitative analysis of the protein level of p-AKT/AKT in the four groups. **C** Quantitative analysis of the protein level of p-PI3K/PI3K in the four groups. **D** Western blotting showing the protein expression of eNOS, p-eNOS and PTEN. **E** Quantitative analysis of the protein level of p-eNOS/eNOS in the four groups. **F** Quantitative analysis of the protein level of PTEN in the four groups. Data are presented as meansSD from three independent experiments. *P<0.05 versus the control (con) group

To investigate the underlying mechanism of the PGZ-Exos salvage of angiogenesis in HUVECs, we used western blotting to detect the expression of PI3K/AKT/eNOS pathway-related proteins. The results revealed that HG decreased the expression of phosphorylated AKT (p-AKT) and phosphorylated PI3K (p-PI3K), while exosomes and PGZ-Exos promoted the expression of p-AKT and p-PI3K. PGZ-Exos had the greatest promoting effect (Fig. 5AC). We found that HG decreased the protein expression of phosphorylated eNOS (p-eNOS). Exosomes and PGZ-Exos significantly alleviated the inhibitory effect of HG on p-eNOS protein expression, while PGZ-Exos showed the greatest mitigation effect. Meanwhile, our results revealed that HG promoted PTEN expression while exosomes and PGZ-Exos significantly decreased the PTEN expression enhanced by HG (Fig. 5DF). Moreover, PGZ-Exos exhibited the strongest suppressive effect in PTEN expression. These results indicate that PGZ-Exos enhance the angiogenic ability of HUVECs through activation of the PI3K/AKT/eNOS pathway.

### LY294002 suppressed PGZ-Exos-induced angiogenesis through inhibition of the PI3K/AKT/eNOS pathway

**Fig. 6:**
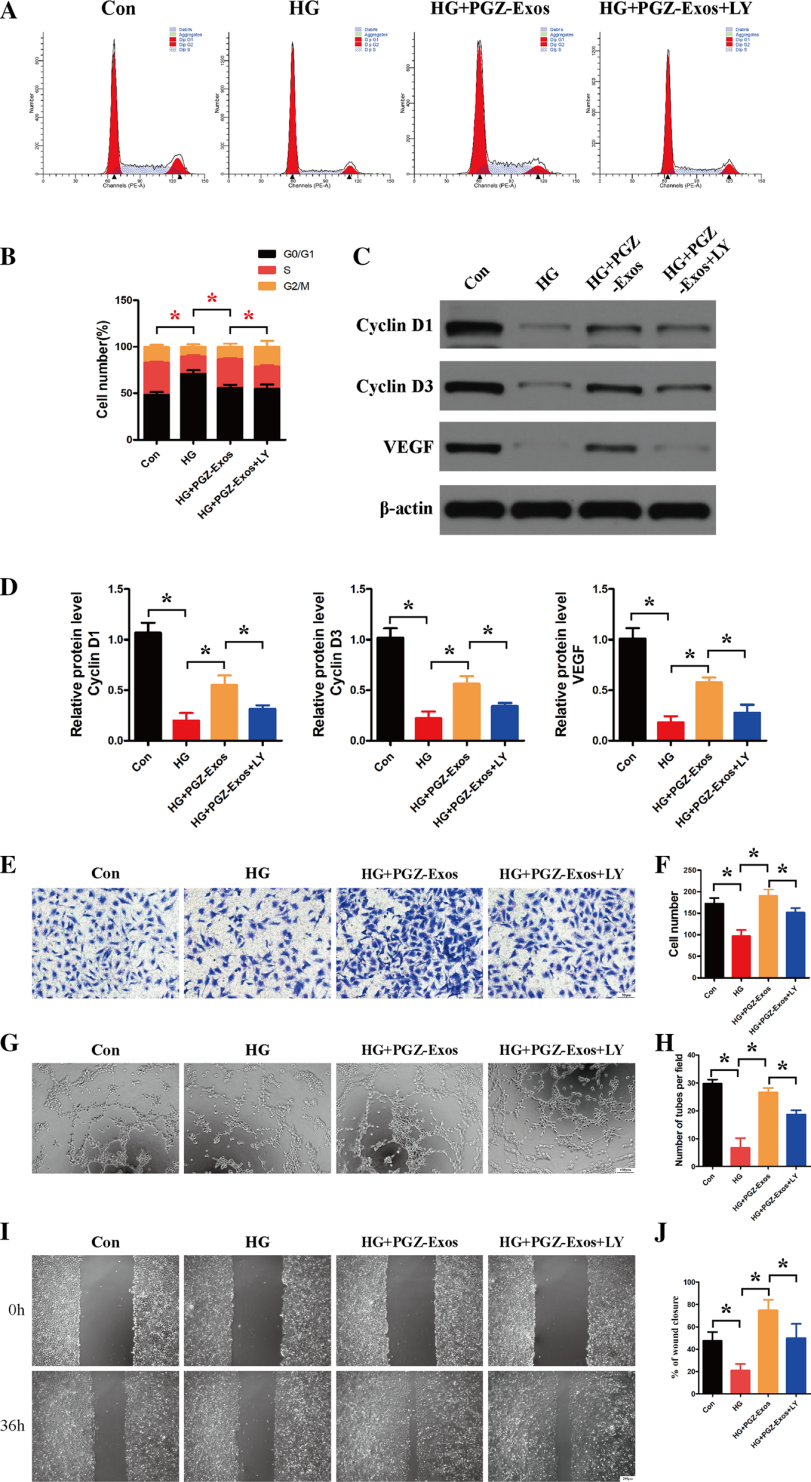
LY294002 suppressed PGZ-Exos-enhanced the biological functions of HUVECs. **A**, **B** Flow cytometry was used to assess the cell cycle distribution of the HUVECs treated with HG medium supplemented with PGZ-Exos and PGZ-Exos+LY 294002. **C**, **D** Western blotting was used to investigate the protein expression of Cyclin D1, Cyclin D3 and VEGF. **E, F** A transwell assay was used to assess the cell migration of HUVECs. Scale bar: 50 m. **G**, **H** A tube formation assay was performed to visualize the cell capillary network formation of HUVECs. Scale bar: 100 m. **I**, **J **In vitro wound healing assay of the HUVECs. Scale bar: 200 m. Data are presented as meansSD from three independent experiments. *P<0.05 versus the control (con) group

**Fig. 7:**
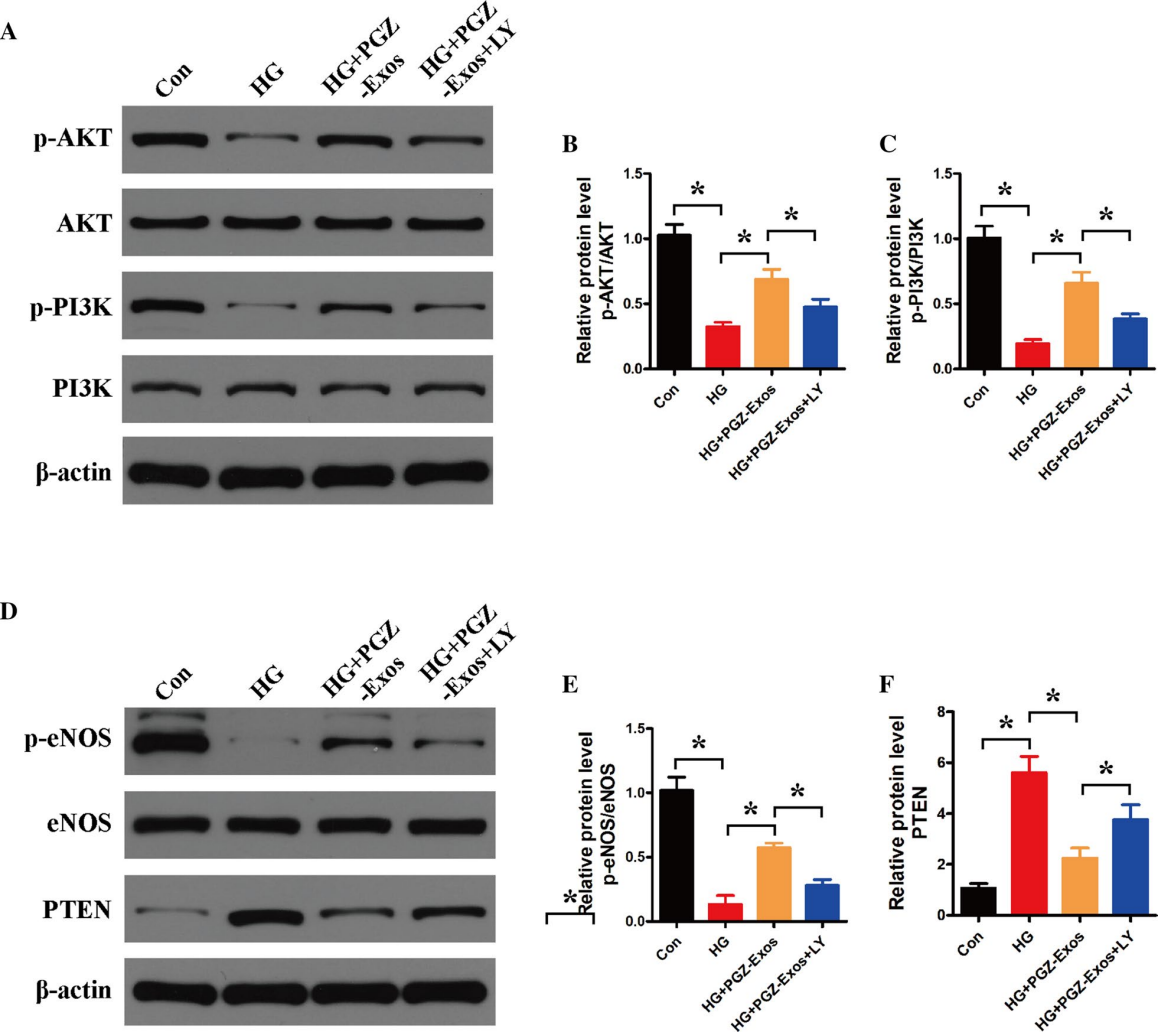
LY294002 inhibited the PGZ-Exos-induced activation of the PI3K/AKT/eNOS pathway in HUVECs. **A** Western blotting showing the protein expression of AKT, p-AKT, PI3K and p-PI3K of the HUVECs treated with HG medium supplemented with PGZ-Exos and PGZ-Exos+LY 294002. **B** Quantitative analysis of the protein level of p-AKT/AKT in the four groups. **C** Quantitative analysis of the protein level of p-PI3K/PI3K in the four groups. **D** Western blotting showing the protein expression of eNOS, p-eNOS and PTEN. **E** Quantitative analysis of the protein level of p-eNOS/eNOS in the four groups. **F** Quantitative analysis of the protein level of PTEN in the four groups. Data are presented as meansSD from three independent experiments. *P<0.05 versus the control (con) group

To further investigate the role of the PI3K/AKT/eNOS pathway in PGZ-Exos induced angiogenesis in HUVECs, we utilized the inhibitor LY294002 to suppress the PI3K/AKT pathway. Flow cytometry demonstrated that LY294002 had inhibitory effects on PGZ-Exos increasing the proportion of cells entering the S phase under hyperglycemic conditions (Fig. 6A, B). LY294002 suppressed the expression of the proliferation-related proteins Cyclin D1 and Cyclin D3 and of VEGF (Fig. 6C, D). Transwell migration assay showed that the HUVECs migration promotion with PGZ-Exos was partially suppressed with LY294002 (Fig. 6E, F). The tube formation ability enhanced by PGZ-Exos was inhibited with LY294002 (Fig. 6G, H). Our results illustrate that LY294002 significantly inhibits the wound healing promoted by PGZ-Exos in vitro (Fig. 6I, J). Western blotting revealed that LY294002 significantly decreased protein expression, including that of p-AKT, p-PI3K and p-eNOS, while increasing PTEN expression (Fig. 7AF). Taken together, these results demonstrated that LY294002 suppressed the PGZ-Exos induced angiogenesis of HUVECs and the PI3K/AKT/eNOS pathway plays an important role in the enhancement of HUVECs angiogenesis by PGZ-Exos.

### PGZ-Exos accelerated diabetic wound healing

**Fig. 8:**
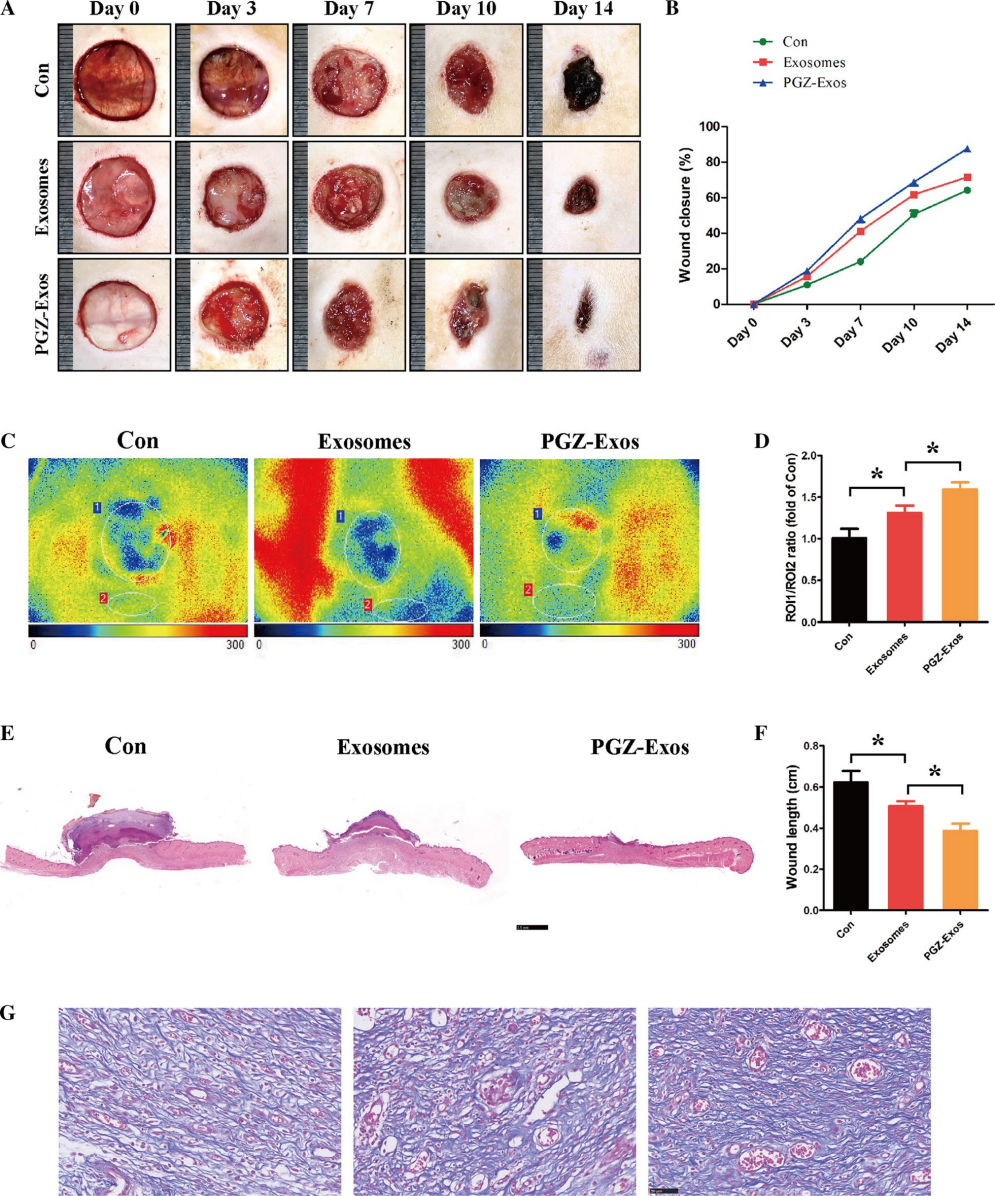
PGZ-Exos accelerated diabetic wound healing. **A** Representative images of full thickness defects in diabetic rats receiving treatment with PBS (Con), exosomes and PGZ-Exo at days 0, 3, 7, 10, and 14 day postoperatively. **B** Wound healing closure rates was calculated among the different groups using the ImageJ software. **C**, **D** Blood perfusion of wounds was assessed with Doppler detection. The results of blood perfusion is presented as the ratio of wound area (ROI-1) to area surrounding the wound (ROI-2). **E**, **F** HE staining and quantification of wound length at day 14. Scale bar: 2.5 mm. **G** Massons trichrome staining at day 14. Scale bar: 50 m

**Fig. 9:**
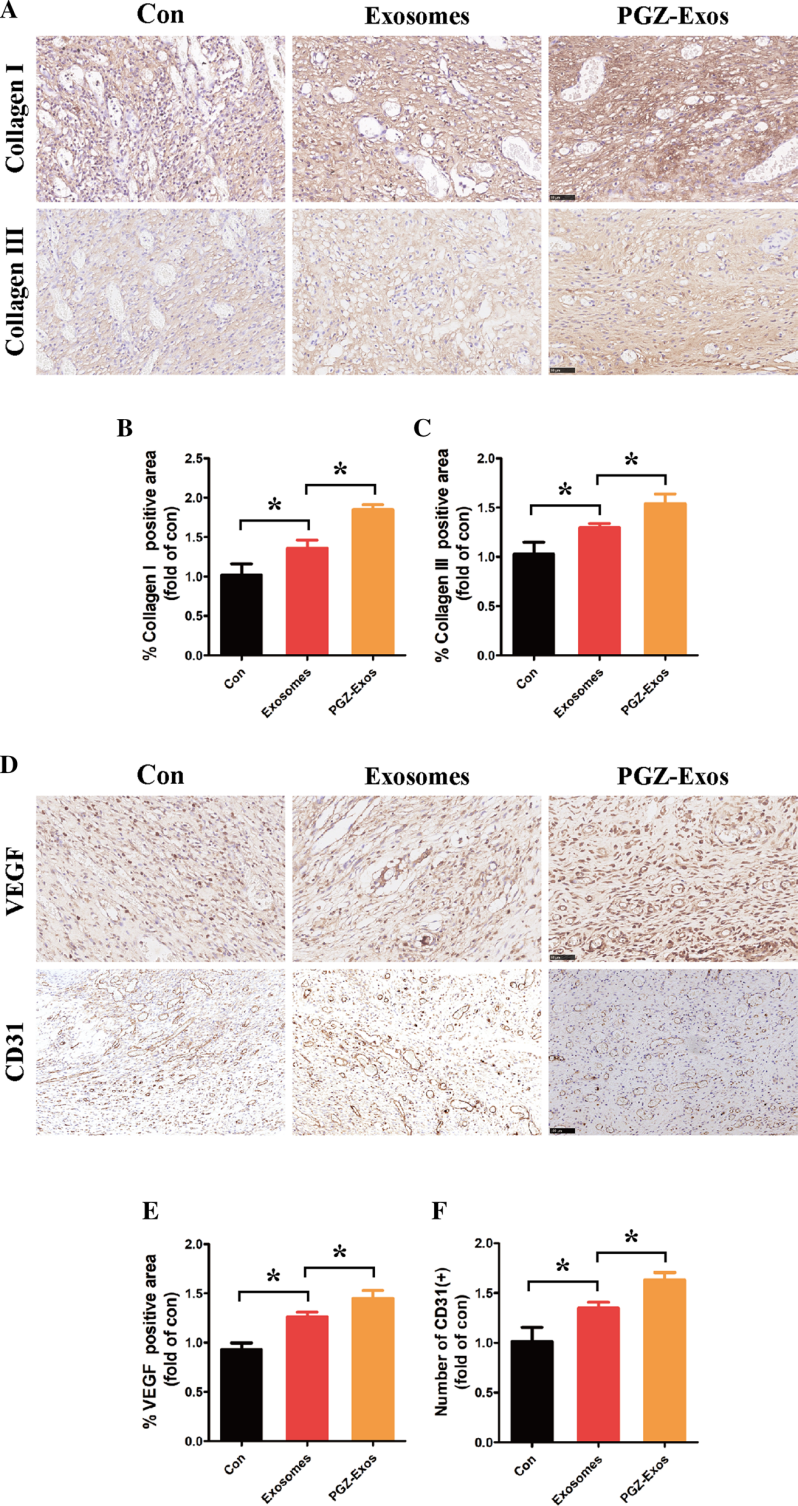
Immunohistochemical analysis of diabetic wounds post-surgery. **A** Representative immunohistochemical analysis images of collagen I, collagen III. Scale bar: 50 m. **B**, **C** Quantification of the positive areas of the collagen I and collagen III among the different groups using the ImageJ software. **D** Representative immunohistochemical analysis images of VEGF (Scale bar: 50 m) and CD31 (Scale bar: 100 m). **E** Quantification of the positive areas of VEGF among the different groups using the ImageJ software. **F** Quantification of the number of CD31 positive among the different groups using the ImageJ software. Data are presented as meansSD from three independent experiments. *P<0.05 versus the control (con) group

To further evaluate the curative effect of PGZ-Exos on diabetic wound healing, we generated diabetic rat models through STZ injection. Subsequently, we established full-thickness dermal dorsal defects and macroscopic assessment of the wounds illustrated that both exosomes and PGZ-Exos accelerated the wound closure compared to the control group. PGZ-Exos showed the greatest effect on days 7, 10, and 14 postoperatively (Fig. 8A, B). Doppler detection to assess perfusion identified that exosomes and PGZ-Exos significantly enhance the blood flow of wound with PGZ-Exos having the greatest effect (Fig. 8C, D). HE staining was used to assess the length and morphology of the wound showing that the diabetic wounds of the PGZ-Exos group had more adequate reepithelization and faster wound closure compared to the control and exosome group (Fig. 8E, F). Masson Trichrome staining demonstrated that more extensive collagen deposition was seen in the PGZ-Exos group, indicating that PGZ-Exos have more superior ECM remodeling ability (Fig. 8G). In addition, immunohistochemistry revealed that PGZ-Exos significantly promoted collagen synthesis, including collagen I and collagen III (Fig. 9AC). PGZ-Exos also increased VEGF production and the expression of CD31 indicating the improvement in blood vessel formation in the diabetic wound healing (Fig. 9DF). These results demonstrate that PGZ-Exos accelerate diabetic wound healing through enhanced angiogenesis.

## Discussion

In this research, we found that PGZ-Exos significantly protect against HG-induced inhibition of HUVECs viability, proliferation and angiogenesis. PGZ-exos enhanced angiogenesis by activating the PI3K/AKT/eNOS pathway. LY294002 inhibited HUVECs function, including proliferation, migration, angiogenesis and wound healing in vitro via inhibition of the PI3K/AKT/eNOS pathway. We further found that PGZ-exos promoted reepithelization, collagen deposition and ECM remodeling in wound healing. These results highlight that PGZ-exos accelerate diabetic wound healing by improving angiogenesis through activation of the PI3K/AKT/eNOS pathway. This provides a promising strategy for improving diabetic wound healing.

It is widely accepted that disruption of local angiogenesis is a crucial limiting factor in diabetic wound healing [5, 27, 36]. Endothelial dysfunction seen with diabetic vascular complications results in impaired repair and abnormal angiogenesis [37]. Increasing evidence has revealed that hyperglycemia-induced impairment of endothelial cell function is an important cause of vascular dysfunction, which results in the occurrence of diabetic complications [38, 39]. Prior studies have demonstrated that HG can induce endothelial cell apoptosis and dysfunction by activating NF-B signaling, leading to diabetic vascular complications [40]. Han et al. reported that HG can suppress cell viability, induce cell apoptosis and cause oxidative stress injury in HUVECs, resulting in a vascular disorder in patients with diabetes [41]. In the present study, our results indicated that HG can inhibit HUVECs viability and proliferation, inducing cellular dysfunction and limiting migration, angiogenesis and wound healing.

MSCs are pluripotent cells with differentiation abilities and immunomodulatory properties, which can significantly enhance the regenerative capacity of many tissues [42, 43]. Studies have suggested that MSCs-secreted angiogenic growth factors can promote bone repair [32] and wound healing [44] through enhanced angiogenesis. However, recent studies have reported that MSCs exert their therapeutic effects in tissue repair mainly through paracrine exosomes [45, 46]. Compared with traditional MSCs-based therapies, exosome-based cell-free therapy avoids the side effects associated with cellular therapy, such as immune rejection and ectopic tissue formation [22, 32]. Exosomes possess stronger biological function due to their higher stability and their plasma membrane rendering them less susceptible to disruption [42, 44]. Recent studies have reported that MSCs-derived exosomes inhibit cell apoptosis and can promote the recovery of the epithelial barrier function to treat acute lung injury [47]. Jia et al. demonstrated that MSCs-derived exosomes improve the proliferation and osteogenic capacity of MSCs to promote bone regeneration in older rats [48]. In addition, MSCs-derived exosomes inhibit LPS-induced nucleus pulposus cell pyroptosis in the repair of intervertebral disc degeneration [49]. In this study, we found that exosomes from BMSCs protect against HG-induced inhibition of HUVECs viability and proliferation, and enhanced HUVECs angiogenic ability.

In recent work researchers have concentrated on enhancing the abilities of exosomes through modifications. Recent studies have reported that exosomes derived from atorvastatin-treated MSCs enhanced cell viability, migration and tube formation of endothelial cells, which significantly improved efficacy of the treatment of acute myocardial infarction [50]. Melatonin-stimulated exosomes can protect mitochondrial function and proliferative potential, and significantly increase the expression of angiogenesis-associated proteins in the treatment of chronic kidney disease [51]. In addition, recent research has reported that pioglitazone can increase cellular proliferation and promote VEGF and collagen production in MSCs. They also found that pioglitazone improved the therapeutic effects of MSCs on tendon repair [52]. In this research, our results revealed that PGZ-Exos significantly improved the biological function of HUVECs including their proliferation, migration, tube formation, and overall wound healing effects which were damaged by HG in vitro. Furthermore, the diabetic rat wound models showed that pioglitazone pretreatment enhanced the therapeutic efficacy of MSCs-derived exosomes and accelerated wound healing by enhancing angiogenesis. In the process of tissue repair, VEGF is considered an important regulator of angiogenesis. VEGF has been proved to induce angiogenesis and the formation of granulation tissue, which promote wound repair [53, 54]. In our research, we found that PGZ-Exos increased VEGF expression and further enhanced the angiogenic ability of HUVECs under HG conditions in vitro. Besides, PGZ-Exos also promoted collagen synthesis, including collagen I and collagen III, increased the expression of VEGF and CD31 indicating improved vascular formation in diabetic wound healing in vivo.

Activation of the PI3K/Akt/eNOS pathway is a crucial process in the stimulation of angiogenesis, including HUVECs viability and tube formation [55]. Nitric oxide (NO) is a vessel active substance secreted by the vascular endothelial system and plays a crucial role in maintaining vascular homeostasis [56]. NO is generated by the endothelial nitric oxide synthase (eNOS). The activation of the PI3K/Akt was able to directly phosphorylate eNOS to increase NO production. In contrast, inhibition of the PI3K/Akt pathway is associated with reducing eNOS phosphorylation and inhibits NO production, which is associated with endothelial cell dysfunction [57]. Previous studies have reported that artemisinin can suppresscell death induced by hydrogen peroxicde and protect HUVECs function from oxidative damage through activation of the PI3K/Akt/eNOS pathway [58]. In this research, we show that PGZ-Exos promote the expression of p-AKT, p-PI3K and p-eNOS, which in turn promote angiogenesis through the PI3K/AKT/eNOS pathway. PGZ-Exos suppressed PTEN expression which is considered a negative regulator of the PI3K/AKT pathway. LY294002 inhibited activation of the PI3K/AKT pathway and suppressed the PGZ-Exos-induced angiogenesis. These data demonstrated that PGZ-Exos enhanced angiogenesis of HUVECs by activating the PI3K/AKT/eNOS pathway.

## Conclusions

In summary, our research highlights that PGZ-Exos can enhance the function and angiogenesis ability of HUVECs. This activation occurs through the upregulation of the PI3K/AKT/eNOS pathway. Enhanced angiogenesis is shown to accelerate rodent diabetic wound healing. Our results offer a promising novel cell-free modality for the treatment of diabetic wounds.

## Methods

### Cell culture

All experiments were approved by the Institutional Animal Care and Use Committee Tongji Medical College, Huazhong University of Science and Technology, China. The BMSCs were cultured from five-week-old Sprague Dawley rats as we previously described [59]. Briefly, the bone marrow from the femurs and tibiae of rats was washed and cultured in a medium containing 10% exosome-depleted fetal bovine serum (Vivacell, Germany). After 24 h, the medium was changed to remove non-adherent cells. The HUVECs were purchased from the Cell Bank of the Chinese Academy of Science (Shanghai, China) and cultured in a complete medium containing 10% fetal bovine serum (FBS, Gibco, USA)_._ To simulate hyperglycemic conditions in vitro, HUVECs were treated with D-(+)-Glucose (Sigma-Aldrich, USA) at 35 mmol/l concentration. A control group (con) was treated with 5.6 mmol/l glucose.

### Isolation and identification of exosomes

To isolate PGZ-Exos, BMSCs were pretreated with 50 M pioglitazone (Selleck Chemicals, USA) in a culture medium containing 10% exosome-depleted fetal bovine serum for 48 h. The culture medium of the BMSCs was then harvested in a sterile environment. PGZ-Exos were isolated from the supernatant via ultracentrifugation. Briefly, the medium of the BMSCs was harvested and centrifuged at 2000*g* for 30 min. The supernatant was centrifuged at 12,000*g* for 45 min. The conditioned medium was filtered using a 0.2 m pore membrane and was centrifuged at 100,000*g* for 90 min. Subsequently, the sediment was resuspended in PBS for further experiments. The morphology of the exosomes was visualized with transmission electron microscopy (Hitachi, Japan). Exosomal markers (CD9 and CD81) were detected by Western blotting. The size distribution of exosomes was measured with Nanoparticle tracking analysis (NTA).

### Exosome uptake

The HUVECs were cultured in a culture plate overnight. Exosomes were pre-labelled with the red fluorescence dye PKH26 (Sigma-Aldrich, USA) according to the manufacturers instructions. The PKH26-labeled exosomes were co-cultured with HUVECs for 6 h. After co-culturing, HUVECs were washed with PBS twice and fixed with 4% paraformaldehyde for 30 min. The cells were stained with FITC Phalloidin (Solarbio, China) for one hour. The cell nuclei were counterstained with Hoechst 33342. After washing with PBS, fluorescence was visualized with a laser scanning confocal microscope (Nikon A1, Japan).

### CCK-8 assay

The cell viability of the HUVECs was detected with a Cell Counting Kit-8 (CCK-8, Dojindo, Japan) assay. Briefly, HUVECs were seeded into 96-well plate and exosomes and PGZ-Exos were added to the culture medium. After treatement, 100 ml CCK-8 solution were added to the 96-well plates. The 96-well plates were incubated at 37 C and the absorbance at 450 nm was detected with a spectrophotometer (BioTek, USA).

### EdU incorporation assay

The cell proliferation of HUVECs was detected with a 5-ethynyl-2-deoxyuridine (EdU) incorporation assay (Ribobio, China) as we previously described [60]. After treatment, the HUVECs were washed twice with PBS. An EdU incorporation assay was used to stain the HUVECs according to the manufacturers instructions. Subsequently, EdU staining images were obtained with a fluorescence microscope (Olympus, Japan).

### Cell cycle

The cell cycle of the HUVECs was evaluated with a cell cycle detection kit (Keygen, China). After treatment, HUVECs were washed twice with PBS and collected with trypsin. After centrifugation, the cells were fixed with 70% cold ethyl alcohol overnight. They were washed with PBS, and the cells were resuspended and incubated with propidium iodide for 30 min in the dark according to manufacturers instructions. Cell cycle distribution was detected with flow cytometry (Becton Dickinson, USA).

### Transwell assay

Migration was assessed with a 24-well transwell system containing 8 m pore-sized filters. Briefly, the HUVECs with serum-free medium were seeded in the upper chamber and exosomes or other reagents were added to the lower chamber. After incubation, the filters were fixed with 4% paraformaldehyde at room temperature. The migrated cells were then stained with crystal violet and observed under an optical microscope (Olympus, Japan).

### Tube formation assay

The capillary network formation of HUVECs was evaluated with a tube formation assay on Matrigel (Corning, USA). Briefly, the cold 96-well plate was loaded with the matrigel at 70 l/well and shaken evenly in ice. After the HUVECs received different treatments, the cells were seeded onto the matrigel-coated plate and incubated with culture medium for 6 h at 37 C. The capillary-like structure formation was observed under an optical microscope (Olympus, Japan) and the number of formed capillaries was counted using ImageJ (version 1.52a; Media Cybernetics, USA).

### Wound healing assay

HUVECs were seeded into six-well plates. After reaching 90% confluence, wounds were created in each well with a sterile 1 ml micropipette tip. The floating cells were washed twice with PBS. The HUVECs received different treatments. Images of each scratch were observed at 0 h and 36 h with an optical microscope (Olympus, Japan). The percentage of wound closure was assessed using ImageJ (version 1.52a; Media Cybernetics, USA).

### Western blot analysis

The proteins of HUVECs were isolated using a RIPA lysis solution (Beyotime, China). After treatment, HUVECs were washed with cold PBS twice and RIPA lysis solution was added to the cells. The protein extracts were harvested at 12,000*g* at 4 C for 10 min. Protein concentration was detected using a BCA protein assay kit (Beyotime, China). Equal amounts of protein were separated by 1012% sodium dodecyl sulfatepolyacrylamide gel electrophoresis and transferred to PVDF membranes (MilliporeSigma, USA). The membranes were blocked with 5% nonfat milk and incubated with the primary antibodies p-AKT (Abcam, UK), AKT (Abcam, UK), PI3K (Abcam, UK), p-PI3K (Abcam, UK), eNOS (Abcam, UK), p-eNOS (Abcam, UK), PTEN (Abcam, UK), and -actin (Abcam, UK) overnight at 4 C. The membranes were washed with PBST thrice and incubated with the appropriate secondary antibody at room temperature for one hour. Subsequently, the proteins of the membranes were visualized according to the manufacturers instructions.

### Animal model

A diabetes model was induced in SpragueDawley rats (SD, 200250 g, male). Briefly, the SD rats were injected with streptozotocin (STZ, 65 mg/kg, Sigma, USA). Rats with a blood glucose level >250 mg/dL were selected for surgery. After the rats were anesthetized with chloral hydrate, a circular full-thickness dermal defect (15 mm diameter) was aseptically created, the rats received a total 100 l PBS, 100 l exosomes (100 g exosomes in 100 l PBS) or 100 l PGZ-Exos (100 g PGZ-Exos in 100 l PBS) injection around the wounds by multisite subcutaneous injection (at least six sites). After injection, an occlusive dressing (Tegaderm; 3M, USA) was applied to cover the wound. Abundant food and water was provided. On post-operative days 0, 3, 7, 10, and 14, the wound was imaged with a digital camera. The diameter of the wound was measured in ImageJ (version 1.52a; Media Cybernetics, USA). After 14 days, the wounds of the rats were harvested and fixed with 4% paraformaldehyde for further assesment.

### Blood perfusion assessment with Doppler detection

The blood flow of the wounds was assessed with Doppler detection on post-operative day 10. Briefly, the rats were anesthetized with chloral hydrate. The mean blood perfusion was detected in the wound area of rats with Doppler detection (ROI 1). Mean blood perfusion was also detected in the surrounding skin, assessed at a standard distance from the wound border (ROI 2). The mean perfusion ratio was counted by comparing the ROI-1value with the ROI-2 value.

### Histological analysis

After fixation with 4% paraformaldehyde, the harvested wounds were dehydrated and embedded in paraffin and the paraffin samples sliced. The sliced samples were dewaxed for haematoxylin and eosin (H&E) and Massons trichrome (MT) staining to visualize the length of the epithelizationand the degree of collagen maturity, respectively. The staining was visualized with an optical microscope. For immunohistochemistry, the paraffin samples were rehydrated. Subsequently, the samples were incubated with the primary antibodies collagen I, collagen III, VEGF and CD31. The paraffin samples were processed with secondary antibodies. Finally, the samples were visualized with the DAB substrate under an optical microscope.

### Statistical analysis

Data are presented as meansSD from at least three separate experiments. The analysis was performed in Graph Pad Prism (GraphPad Software Inc; La Jolla, CA, USA). Students t-test was used to compare differences between two groups. One-way ANOVA, followed by Tukeys multiple comparison test was used to analyze differences in more than two groups. P<0.05 was considered statistically significant.

## Data Availability

All data generated or analyzed during this study are included in this published article.
